# Automatic Segmentation of the Fetus in 3D Magnetic Resonance Images Using Deep Learning: Accurate and Fast Fetal Volume Quantification for Clinical Use

**DOI:** 10.1007/s00246-022-03038-0

**Published:** 2022-11-05

**Authors:** Daniel Ryd, Amanda Nilsson, Einar Heiberg, Erik Hedström

**Affiliations:** 1 Clinical Physiology, Department of Clinical Sciences Lund, Lund University, Skane University Hospital, Lund, Sweden; 2 Wallenberg Centre for Molecular Medicine, Lund University, Lund, Sweden; 3 Diagnostic Radiology, Department of Clinical Sciences Lund, Lund University, Skane University Hospital, Lund, Sweden

**Keywords:** Fetal cardiovascular magnetic resonance imaging, Fetal weight, Prenatal diagnosis

## Abstract

Magnetic resonance imaging (MRI) provides images for estimating fetal volume and weight, but manual delineations are time consuming. The aims were to (1) validate an algorithm to automatically quantify fetal volume by MRI; (2) compare fetal weight by Hadlock’s formulas to that of MRI; and (3) quantify fetal blood flow and index flow to fetal weight by MRI. Forty-two fetuses at 36 (29–39) weeks gestation underwent MRI. A neural network was trained to segment the fetus, with 20 datasets for training and validation, and 22 for testing. Hadlock’s formulas 1–4 with biometric parameters from MRI were compared with weight by MRI. Blood flow was measured using phase-contrast MRI and indexed to fetal weight. Bland–Altman analysis assessed the agreement between automatic and manual fetal segmentation and the agreement between Hadlock’s formulas and fetal segmentation for fetal weight. Bias and 95% limits of agreement were for automatic versus manual measurements 4.5 ± 351 ml (0.01% ± 11%), and for Hadlock 1–4 vs MRI 108 ± 435 g (3% ± 14%), 211 ± 468 g (7% ± 15%), 106 ± 425 g (4% ± 14%), and 179 ± 472 g (6% ± 15%), respectively. Umbilical venous flow was 406 (range 151–650) ml/min (indexed 162 (range 52–220) ml/min/kg), and descending aortic flow was 763 (range 481–1160) ml/min (indexed 276 (range 189–386) ml/min/kg). The automatic method showed good agreement with manual measurements and saves considerable analysis time. Hadlock 1–4 generally agree with MRI. This study also illustrates the confounding effects of fetal weight on absolute blood flow, and emphasizes the benefit of indexed measurements for physiological assessment.

## Introduction

Congenital anomalies and fetal growth restriction are major contributors to fetal and neonatal morbidity and mortality [1, 2]. Prenatal diagnosis improves management including parental counseling [3–8]. Accurate quantification of fetal volume and hence fetal weight is important both for assessment of fetal growth and for indexing fetal blood flow to fetal weight. This provides a physiologically more accurate interpretation of blood flow volumes as blood flow is related both to fetal body size and pathology. Indexed umbilical blood flow volumes may also be a potential indicator of fetal growth restriction and placental dysfunction [9].

In clinical practice, fetal weight is estimated by ultrasound [10–12], and while these methods are widely available and easy to use, they are less accurate than 3D-based fetal segmentation [13].

Magnetic resonance imaging (MRI) can provide high-resolution 3D images for fetal volume quantification. However, accurate manual segmentation of the fetus is time consuming, and there is a need for fully automatic methods to make quantification of fetal volume and weight clinically applicable. Deep learning could potentially be used to accomplish automatic and fast fetal volume quantification [14, 15]. However, previous studies have either shown significant measurement errors for automatic versus manual segmentation or involved network structures that require highly specialized graphics cards.

The aims of this study were therefore to (1) validate an algorithm based on an artificial neural network to automatically quantify fetal volume from 3D MRI; (2) compare fetal weight estimated using formulas commonly used in fetal ultrasound with 3D MRI-based fetal weight measurements; and (3) quantify fetal blood flow in the umbilical vein and descending aorta and index blood flow to fetal weight by MRI.

## Methods

Forty-two fetuses (gestational age 36 (29–39) weeks) underwent fetal MRI at Skane University Hospital in Lund, Sweden between October 2015 and December 2021. Fetal MRI examinations were performed both on clinical indication as dedicated fetal cardiovascular MRI to assess fetal cardiovascular anatomy, and for research aimed at developing fetal cardiovascular MRI. The cohort consisted of fetuses with and without known or suspected congenital heart disease. The regional ethics committee approved the study (Dnr 2013/551). All pregnant women gave written informed consent before participation in the study. The study was conducted in accordance with the Helsinki declaration.

### Magnetic Resonance Image Acquisition

Fetal MRI was performed using a 1.5 T Aera scanner (Siemens Healthineers, Erlangen, Germany). Balanced steady-state free precession (bSSFP) sequences were used to acquire anatomical overview images in the transverse, sagittal, and coronal directions with typical parameters 1.7 × 1.1 × 4.5 mm acquired spatial resolution and a slice gap of 0 or −50%. For fetal volume quantification, a 3D image slab covering the uterus was acquired with typical parameters 1.8 × 1.4 × 2.5 mm acquired resolution, TE/TR = 1.77/4.08 ms, and flip angle = 50°. Phase-contrast flow images were acquired in the umbilical vein and fetal descending aorta using a 2D gradient recalled echo sequence with typical parameters 1.4 × 1.4 × 5 mm acquired spatial resolution, TE/TR = 2.76/5.03 ms, flip angle = 20°, VENC = 150 cm/s, and acquired temporal resolution 30.18 ms. The fetal MRI examination time was typically 40–60 min including research and development, whereas the 3D acquisition is less than 20 s.

### Magnetic Resonance Image Analysis

**Fig. 1:**
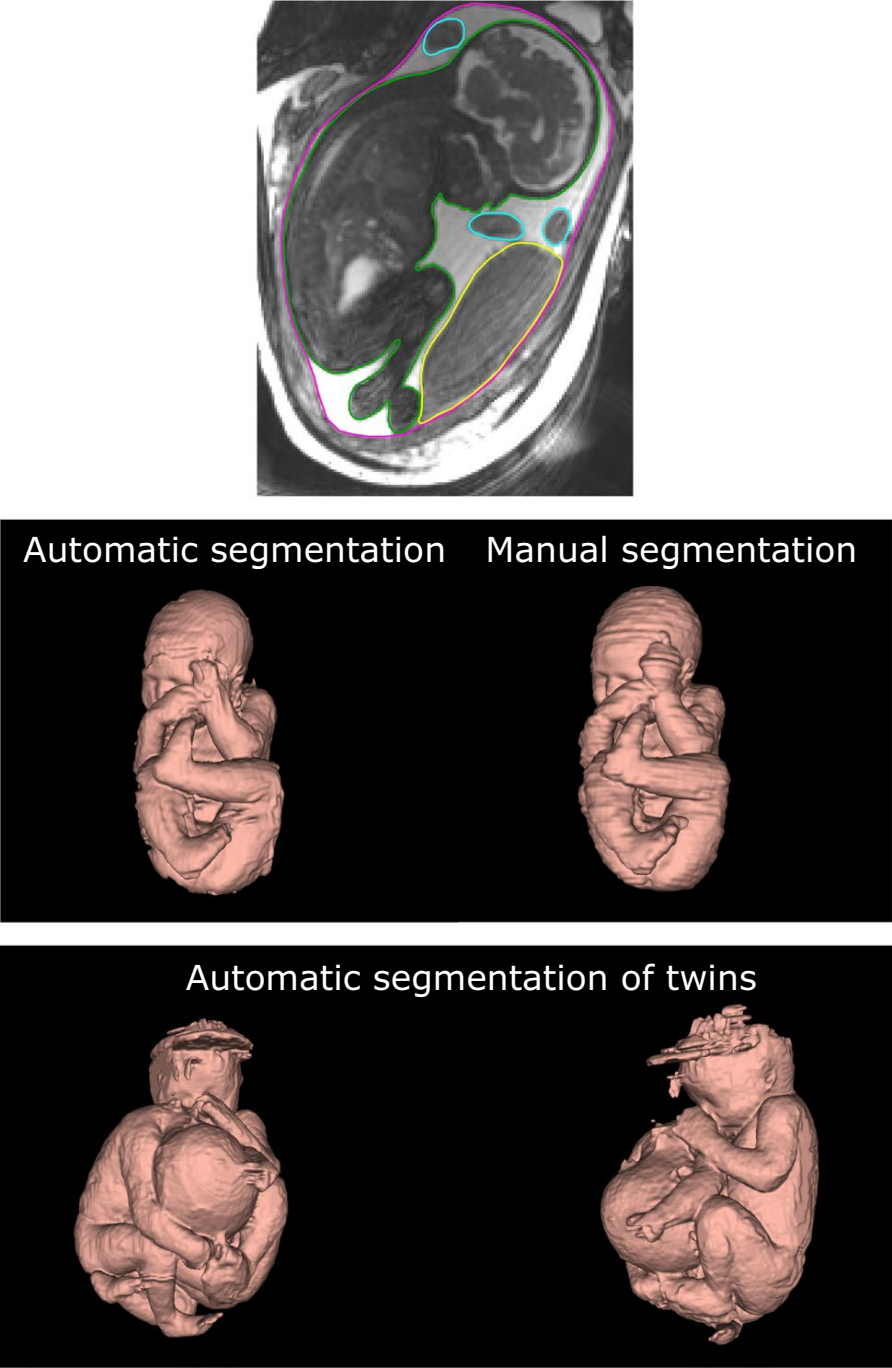
Segmentation of the fetus in magnetic resonance images. The top panel shows a magnetic resonance image with manual delineations of the fetus (green), placenta (yellow), umbilical cord (blue), and uterine wall (pink). This was repeated throughout the 3D image stack and all pixels in the image stack were classified as fetus, placenta, umbilical cord, or amniotic fluid. This pixel classification was used for training and evaluation of the proposed artificial neural network. The middle panel shows fetal 3D models generated by automatic (left) and manual (right) segmentation of the same fetus. The time required to generate the automatic model is 45 s, whereas the time required to generate an accurately manually segmented model is 1–2 h. Agreement between manual and automatic fetal segmentation is high (c.f. Fig. 3). The bottom panel shows the performance of the automatic method on twin fetuses. The proposed automatic fetal segmentation method was tested on a case of twin fetuses as proof of concept to show generalizability. Although the algorithm had only been trained on singleton fetuses, it shows promising generalizability. Artifacts at the top of one of the fetal heads are related to image artifacts in the 3D MRI images

Manual segmentations of the fetus, umbilical cord, placenta, and amniotic fluid were performed in Segment 3D print v 3.1 (Medviso AB, Lund, Sweden) using a 3D pen tool with a diameter of 3–4 mm (Fig. 1; top panel). Manual delineations were used as ground truth for training of neural networks and for evaluation of network performance. Fetal weight was calculated as fetal volume multiplied with a fetal density of 1.04 kg/l, as previously reported in late gestation fetuses [13].

**Fig. 2:**
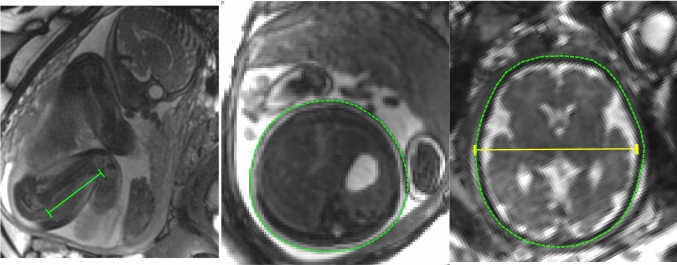
Fetal biometry for using Hadlock’s weight estimation formulas. Three typical images used for measurements of fetal femur length (green; left), abdominal circumference (green; middle), head circumference (green; right) and biparietal diameter (yellow; right). Femur length was measured in the anatomical overview images, as the fetal femur was generally not visible in the 3D images due to low contrast, whereas the other measurements were performed on 3D data after multiplanar reformatting

Fetal weight was also estimated using Hadlock’s formulas 1–4 [11, 12] for direct comparison of accuracy of ultrasound-based measurements versus 3D MRI fetal weight as reference standard. For this, biometric parameters were measured in MR images. Figure 2 shows how these measurements of fetal head circumference (HC), biparietal diameter (BPD), abdominal circumference (AC) and femur length (FL) were performed. In the current study, the following Hadlock formulas were used; Hadlock 1: ${Log}_{10}\left(weight\right)= 1.304+0.05281\bullet \mathrm{AC}+0.1938\bullet \mathrm{FL }-0.004\bullet \mathrm{AC}\bullet \mathrm{FL}$; Hadlock 2: ${Log}_{10}\left(weight\right) = 1.335-0.0034\bullet \mathrm{AC}\bullet \mathrm{FL}+ 0.0316\bullet \mathrm{BPD}+0.0457\bullet \mathrm{AC }+0.1623\bullet \mathrm{FL}$; Hadlock 3: ${Log}_{10}\left(weight\right)=1.326-0.00326 \bullet \mathrm{AC}\bullet \mathrm{FL}+0.0107\bullet \mathrm{HC }+0.0438\bullet \mathrm{AC }+ 0.158\bullet \mathrm{FL}$; Hadlock 4: ${Log}_{10}\left(weight\right)=1.3596 -0.00386\bullet \mathrm{AC}\bullet \mathrm{FL}+0.0064\bullet \mathrm{HC}+0.00061\bullet \mathrm{BPD}\bullet \mathrm{AC}+ 0.0424\bullet \mathrm{AC}+0.174\bullet \mathrm{FL}$.

Blood flow was quantified in the umbilical vein in 15 fetuses and in the fetal descending aorta in 20 fetuses by manual vessel delineation using Segment v3.3 (Medviso AB, Lund, Sweden) [16, 17].

### Algorithm for Automatic Fetal Segmentation

The algorithm was developed in a previous project [18]. In short, a core part of the algorithm is a U-net convolutional neural network [18, 19] trained to classify each pixel as fetus, placenta, umbilical cord, or amniotic fluid with manual delineations as ground truth [18]. While the fetus was the object of interest in the current study, the inclusion of all intrauterine structures was used in a multi-task learning process to provide more information to the network to improve network performance [20]. In contrast to previous attempts to automatically segment the fetus [15], the current network structure is a 2D U-net which processes data on a slice-by-slice basis in three different orthogonal directions. The final segmentation result is thus a voxel-wise voting of the three directions. Fourfold cross-validation was used for training and hyperparameter optimization, with 15 datasets used for training and 5 for validation for each iteration. Of the remaining 22 datasets, 21 were used for testing network performance versus manual segmentation, and one twin pregnancy dataset was used to test generalizability of the network as proof of concept.

### Statistics

Fetal volumes and weights are reported in milliliters and grams, respectively. Bland–Altman analysis was used to assess agreement between automatic and manual fetal volume measurements, and between fetal weight estimated using Hadlock’s formulas and by volumes from 3D MRI. In addition to Bland–Altman analysis, agreement between automatic and manual fetal segmentation was assessed using the Dice similarity coefficient, defined for two sets A and B as $\frac{2\cdot |A\cap B|}{\left|A\right|+|B|}$ and expressed as mean ± standard deviation. Absolute blood flow and blood flow indexed to fetal weight were plotted against fetal weight to illustrate the confounding effects of fetal weight on absolute blood flow and to provide proof-of-concept data on indexed blood flow values using the proposed method.

## Results

**Fig. 3:**
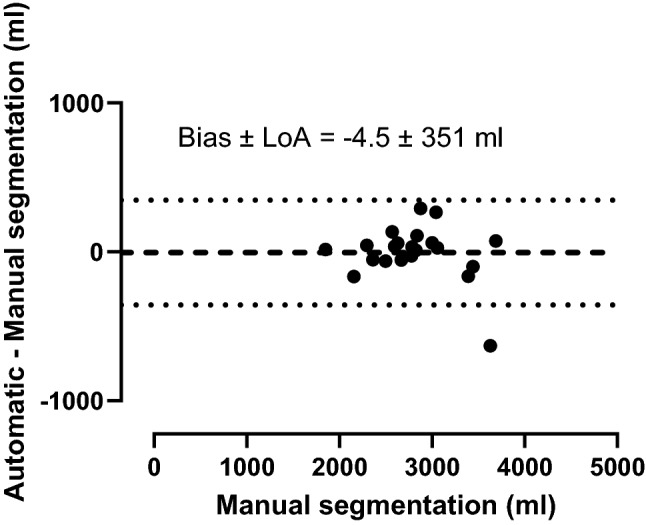
Bland–Altman analysis for agreement between automatic and manual fetal volume measurements. Dashed lines indicate bias and dotted lines indicate 95% limits of agreement (LoA)

Figure 1 (middle panel) shows an example of 3D fetal models generated by automatic and manual segmentation. This shows a visual good agreement between the automatic and manual fetal segmentation. Figure 3 shows the agreement between automatic and manual fetal volumes for the test set. Bias and 95% limits of agreement were −4.5 ± 351 ml (0.01% ± 11%). Mean Dice similarity index for automatic versus manual fetal segmentation was 0.94 ± 0.02.

**Fig. 4:**
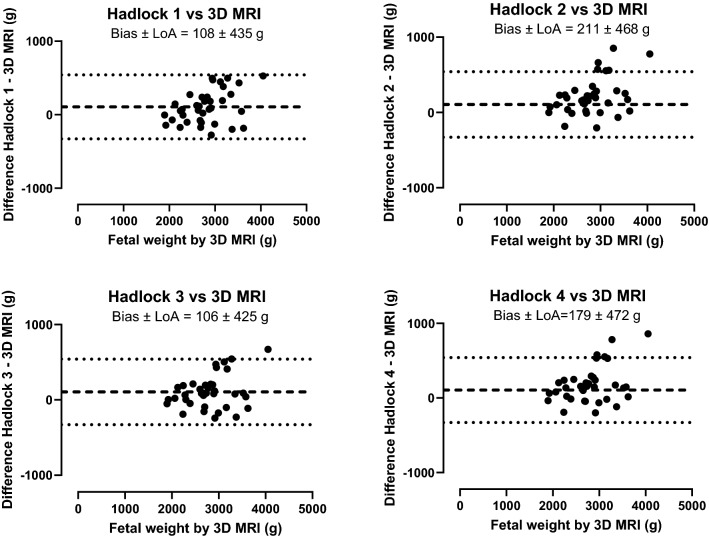
Bland–Altman analysis for fetal weight by Hadlock’s formulas versus 3D MRI. Fetal weight estimated by Hadlock’s formulas 1–4 agrees with fetal weight based on 3D MRI measurements, however with wide limits of agreement There is however a trend of increasing differences with increasing fetal weight. Dashed lines indicate bias and dotted lines indicate 95% limits of agreement (LoA)

Figure 4 shows the agreement between Hadlock’s formulas 1–4 for fetal weight estimation and fetal weight by 3D MRI. Bias and 95% limits of agreement for Hadlock’s formulas 1–4 versus 3D MRI manual delineations were 108 ± 435 g (3% ± 14%), 211 ± 468 g (7% ± 15%), 106 ± 425 g (4% ± 14%), and 179 ± 472 g (6% ± 15%), respectively. Weight estimation by Hadlock’s formulas showed a trend of increasing differences versus 3D MRI with increasing fetal weight.

Figure 1 (bottom panel) shows the result of the proposed automatic method applied on twin fetuses as proof of concept of using the network on other samples than the singleton pregnancies included for evaluation versus manual segmentation.

**Fig. 5:**
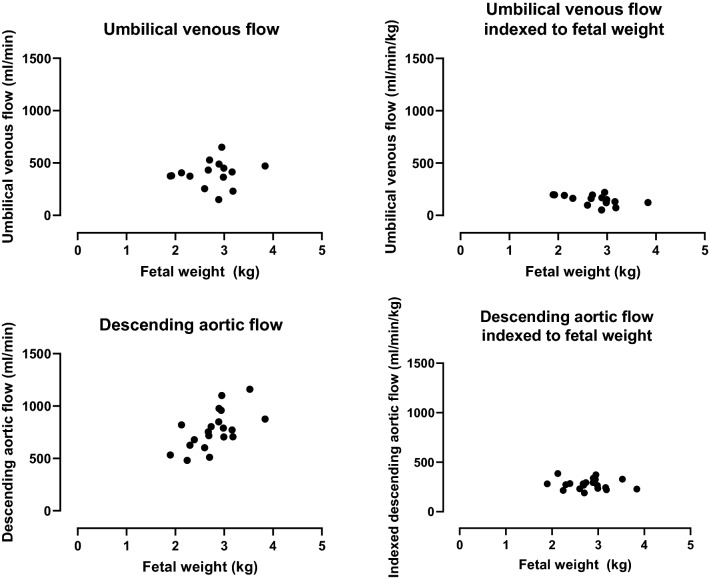
Absolute fetal blood flow and blood flow indexed to fetal weight. Blood flow versus fetal weight in the umbilical vein (top panel) and fetal descending aorta (bottom panel) for absolute flow volumes (left) and flow volumes indexed to fetal weight (right). Indexed blood flow volumes enable physiological comparisons between fetuses so as to study pathophysiology

Figure 5 shows blood flow in the umbilical vein and descending aorta in absolute and indexed values. Median absolute umbilical venous flow was 406 ml/min (range 151–650 ml/min), which indexed to fetal weight was 162 ml/min/kg (range 52–220 ml/min/kg). Median absolute descending aortic flow was 763 ml/min (range 481–1160 ml/min), which indexed to fetal weight was 276 ml/min/kg (range 189–386 ml/min/kg).

## Discussion

This study validated an automated deep learning-based algorithm for automatic quantification of fetal volume and weight from MR images. The automatic method showed high accuracy for fetal volume measurements compared with manual reference standard. While manual adjustments may be needed in some cases, the automatic method typically takes 45 s per fetus and therefore saves considerable analysis time per case. The automatic method thus makes it feasible to accurately quantify fetal volume and weight for both clinical and research purposes. This may lead to more accurate assessment of fetal growth and to improved assessment of fetal blood flow by indexing flow volumes to fetal weight, as absolute blood flow is dependent on the size of the fetus and not only pathology.

The current study showed a generally good agreement between Hadlock’s formulas and 3D MRI-based fetal segmentation for estimation of fetal weight, however with a trend of increasing differences with increasing fetal size. In particular, this study shows that MRI-based measurements of fetal biometric parameters may be used for accurate weight estimation using Hadlock’s formulas if complete 3D MRI datasets are not available. It remains unknown to what extent such fetal biometric measurements by MRI and ultrasound agree, however, a potential advantage by MRI is the ability to accurately acquire images perpendicular to the fetal head and abdomen and visualize the fetal femur regardless of acoustic windows. Therefore, the results in the current study may have exaggerated agreement in comparison to if fetal weight by MRI and ultrasound had been compared head-to-head. Although Hadlock’s formulas show higher overall accuracy compared to other ultrasound-based weight estimation formulas [10], Hadlock’s formulas are less reliable for small and large fetuses [10]. This may partly explain the trend of increasing differences for Hadlock’s formulas vs 3D MRI with increasing fetal weight observed in the current study. Furthermore, this difference could potentially mean the difference between small for gestational age versus normal weight, and therefore could be clinically significant in individual cases. Thus, the need for improved methods of fetal weight estimation remains and it may be hypothesized that 3D MRI-based fetal weight estimation could improve accuracy and thus clinical decision-making.

The current study agrees with previous studies in that indexed flow volumes may be a more appropriate measure of fetal circulatory physiology than absolute flow volumes, thus enabling accurate comparison of physiology between fetuses independent of fetal weight. Furthermore, the weight-indexed blood flow values obtained by phase-contrast MRI in the current study are in agreement with previously reported weight-indexed fetal blood flow values from ultrasound measurements [21, 22] and by fetal MRI [23].

Two previous studies have suggested machine learning methods for automatic segmentation of the fetus in magnetic resonance images [14, 15]. Zhang et al. (14) used a graph-based approach to automatically segment the fetal body in fetuses at 20–24 weeks of gestation. In the current study, the performance of the automatic method was higher as shown by the Dice similarity index of 0.94 versus 0.69 [14]. However, the current study included mainly late gestation fetuses, and the performance of the proposed method earlier in pregnancy remains to be investigated. In comparison, Dudovitch et al. [15] evaluated both two- and three-dimensional U-nets for automatic segmentation of the fetus, which showed high accuracy (Dice similarity index up to 0.96). No apparent improvement was however seen using a standard three-dimensional U-net compared to a standard two-dimensional U-net. Network performance increased for the three-dimensional U-net with the addition of another network to correct segmentation in slices prone to error. However, that three-dimensional U-net requires advanced graphics cards generally not available in clinical routine settings. Further, the current study used a two-dimensional U-net analyzing the image slab in three orthogonal directions. This means that the decision to classify a pixel as fetus is based on more information compared with standard two-dimensional U-nets, increasing network performance. Furthermore, two-dimensional U-nets are easily implemented on current clinical systems, making the proposed method useful for clinical application.

Finally, ultrasound may be better than 3D MRI for fetal volume quantification in early pregnancy due to the higher resolution of ultrasound images. Although there is currently no automatic method for generating fetal 3D models from ultrasound images, such methods are developing [24, 25]. On the other hand, it may be challenging to get ultrasound images of sufficient quality for segmentation of the whole fetus, particularly in late pregnancy where acoustic windows may be a limiting factor [25, 26]. It is thus plausible that ultrasound and MRI could complement one another to achieve accurate fetal volume quantification in early and late gestation, respectively.

This study has suggested an artificial intelligence-based automatic method for estimation of fetal weight using 3D MRI. This enables routine accurate and fast weight estimation of fetuses undergoing MRI examinations, and therefore adds potentially clinically useful information to existing fetal MRI imaging protocols. Future studies are warranted to develop artificial intelligence-based methods for automatic detection of fetal pathology, such as congenital heart disease, diaphragmatic hernia, or myelomeningocele.

## Limitations

The current study included relatively large fetuses with a weight span of approximately 2000–4000 g. It remains to be shown if fetal volume measurements by the proposed method are feasible in smaller fetuses in early gestation, and to what extent such measurements agree with ultrasound-based estimations. However, in order to test the generalizability of the network, the automatic segmentation algorithm was tested on a twin pregnancy with promising results despite that the algorithm was not trained in twin pregnancies. This shows strong potential for the proposed method to work across a wider range of fetal sizes.

## Conclusions

The proposed method can be clinically applied for automatic segmentation of fetal volume and weight. This saves analysis time and makes indexation of fetal blood flow to fetal size clinically feasible. Further, it could be a useful complement in clinical practice for assessing fetal growth restriction, particularly when acoustic windows are poor as in late gestation fetuses and in fetuses suspected to be smaller or larger than what standard ultrasound methods are accurate for. Indexed fetal blood flow values were similar across the range of fetal weights in the current study, which illustrates the confounding effect of fetal weight and the benefit of indexed values for physiological comparison between individuals.

## References

[1] Garite TJ, Clark R, Thorp JA (2004). Intrauterine growth restriction increases morbidity and mortality among premature neonates. Am J Obstet Gynecol.

[2] Boyle B, Addor MC, Arriola L, Barisic I, Bianchi F, Csáky-Szunyogh M (2018). Estimating global burden of disease due to congenital anomaly: an analysis of european data. Arch Dis Child Fetal Neonatal Ed.

[3] Levery A, Glickstein JS, Kleinman CS, Levasseur SM, Chen J, Gersony WM (2010). The Impact of prenatal diagnosis of complex congenital heart disease on neonatal outcomes. Pediatr Cardiol.

[4] Satomi G, Yasukochi S, Shimizu T, Takigiku K, Ishii T (1999). Has fetal echocardiography improved the prognosis of congenital heart disease? Comparison of patients with hypoplastic left heart syndrome with and without prenatal diagnosis. Pediatr Int.

[5] Chakraborty A, Gorla SR, Swaminathan S (2018). Impact of prenatal diagnosis of complex congenital heart disease on neonatal and infant morbidity and mortality. Prenat Diagn.

[6] Holland BJ, Myers JA, CRW. (2015). Prenatal diagnosis of critical congenital heart disease reduces risk of death from cardiovascular compromise prior to planned neonatal cardiac surgery: a meta-analysis. Ultrasound Obstet Gynecol.

[7] Franklin O, Burch M, Manning N, Sleeman K, Gould S, Archer N (2002). Prenatal diagnosis of coarctation of the aorta improves survival and reduces morbidity. Heart.

[8] Allan LD, Huggon IC (2004). Counselling following a diagnosis of congenital heart disease. Prenat Diagn.

[9] Ferrazzi E, Rigano S, Bozzo M, Bellotti M, Giovannini N, Galan H (2000). Umbilical vein blood flow in growth-restricted fetuses. Ultrasound Obstet Gynecol.

[10] Hammami A, Mazer Zumaeta A, Syngelaki A, Akolekar R, Nicolaides KH (2018). Ultrasonographic estimation of fetal weight: development of new model and assessment of performance of previous models. Ultrasound Obstet Gynecol.

[11] Hadlock FP, Harrist RB, Sharman RS, Deter RL, Park SK (1985). Estimation of fetal weight with the use of head, body, and femur measurements: a prospective study. Am J Obstet Gynecol.

[12] Hadlock FP, Harrist RB, Carpenter RJ, Deter RL, Park SK (1984). Sonographic estimation of fetal weight. The value of femur length in addition to head and abdomen measurements. Radiology.

[13] Kacem Y, Cannie MM, Kadji C, Dobrescu O, Lo ZL, Ziane S (2013). Fetal weight estimation: comparison of two-dimensional US and MR imaging assessments. Radiology.

[14] Zhang T, Matthew J, Lohezic M, Davidson A, Rutherford M, Rueckert D, et al (2016) Graph-based whole body segmentation in fetal MR images. MICCAI Work PIPPI.

[15] Dudovitch G, Link-Sourani D, Ben Sira L, Miller E, Ben Bashat D, Joskowicz L, Martel AL, Abolmaesumi P, Stoyanov D, Mateus D, Zuluaga MA, Zhou SK (2020). Deep learning automatic fetal structures segmentation in MRI scans with few annotated datasets. Medical image computing and computer assisted intervention: MICCAI 2020.

[16] Heiberg E, Sjögren J, Ugander M, Carlsson M, Engblom H, Arheden H (2010). Design and validation of Segment–freely available software for cardiovascular image analysis. BMC Med Imaging.

[17] Salehi D, Sun L, Steding-ehrenborg K, Bidhult S, Kording F, Ruprecht C (2019). Quantification of blood flow in the fetus with cardiovascular magnetic resonance imaging using Doppler ultrasound gating: validation against metric optimized gating. J Cardiovasc Magn Reson.

[18] Nilsson A (2021) Quantification of fetal volume in magnetic resonance images using deep learning. Student Paper. Master’s Theses in Mathematical Sciences, Faculty of Engineering, Lund University. http://lup.lub.lu.se/student-papers/record/9043734

[19] Ronneberger O, Fischer P, Brox T, Navab N, Hornegger J, Wells WM, Frangi AF (2015). U-net: convolutional networks for biomedical image segmentation. Medical image computing and computer-assisted intervention:MICCAI 2015.

[20] Navarro F, Shit S, Ezhov I, Paetzold J, Gafita A, Peeken JC (2019). Shape-aware complementary-task learning for multi-organ segmentation. Lect Notes Comput Sci.

[21] Maršál K, Lindblad A, Lingman G, Eik-Nes SH (1984). Blood flow in the fetal descending aorta; intrinsic factors affecting fetal blood flow, i.e. fetal breathing movements and cardiac arrhythmia. Ultrasound Med Biol.

[22] Figueras F, Fernández S, Hernández-Andrade E, Gratacós E (2008). Umbilical venous blood flow measurement: accuracy and reproducibility. Ultrasound Obstet Gynecol.

[23] Seed M, Van Amerom JFP, Yoo SJ, Al Nafisi B, Grosse-Wortmann L, Jaeggi E (2012). Feasibility of quantification of the distribution of blood flow in the normal human fetal circulation using CMR: a cross-sectional study. J Cardiovasc Magn Reson.

[24] Li Y, Xu R, Ohya J, Iwata H (2017) Automatic fetal body and amniotic fluid segmentation from fetal ultrasound images by encoder-decoder network with inner layers. Proc Annu Int Conf IEEE Eng Med Biol Soc 10.1109/EMBC.2017.8037116 29060160

[25] Tutschek B (2018). 3D prints from ultrasound volumes. Ultrasound Obstet Gynecol.

[26] Schild RL (2007). Three-dimensional volumetry and fetal weight measurement. Ultrasound Obstet Gynecol.

